# Book Review: Python Programming for Biology

**DOI:** 10.3389/fgene.2016.00066

**Published:** 2016-04-26

**Authors:** Alberto Marin-Sanguino

**Affiliations:** Specialty Division for Systems Biotechnology, Faculty for Mechanical Engineering, Technical University of MunichGarching, Germany

**Keywords:** Python, systems biology, computational biology, bioinformatics, programming languages

Python is an excellent language for scientists and can be used at many levels: Simple scripts for quick and dirty calculations, big programs implementing complex data models, taking advantage of its powerful libraries for number crunching or simply as “glue” to bind together more specialized modules written in C or Fortran. Such a versatility may be an obstacle to find an entry point for the newcomer. Stevens and Boucher (2015) take computational Biology in the widest sense of the term to write an extensive introduction to Python and provide an overview of its main scientific libraries.

The reader will find a gentle but terse introduction covering the basics in seventy pages before moving on to practical topics like reading relevant file formats (FASTA, pdb, …) From that point on, programming concepts like data models or object orientation are introduced through practical examples. The rest of the book is composed by an assortment of chapters that cover a wide diversity of topics and can be read independently or used as a reference. Some of these chapters are focused on specific biological topics such as macromolecular structures, sequence alignments, array data and high-throughput sequencing. The relevant libraries are introduced along one or more of these chapters to get the user up to speed. Other chapters are centered on a technique or discipline such as image processing, machine learning, probability or statistics. In all cases, a brief introduction on the topic is followed by a series of examples on how to get started. The selection of topics offers a good compromise, presenting the general principles as well as useful recipes without overwhelming the reader with excessive detail. Getting the reader up to speed in a very short time seems to be the key premise. Finally, advanced topics like parallelization and interfacing with C will point the reader to the next level. The book also provides a good overview of the main libraries with immediate applications to biology, although some readers may miss a chapter on pandas.

This book is a good choice for researchers who want to migrate to Python or Ph.D. students about to get started a computational biology or bioinformatics project. Biologists without programming experience may prefer to start with a more gentle and maybe shorter introduction, but those with previous experience with software packages like MATLAB or R will also find this book to be a fast lane to Python. Clear explanations of the biological background will also make this book accessible to scientists intending to move into biology from other disciplines.

## Author contributions

The author confirms being the sole contributor of this work and approved it for publication.

## Funding

AMS acknowledges funding from the German Federal Ministry of Education and Research (BMBF), e:Bio initiative 0316197.

### Conflict of interest statement

The author declares that the research was conducted in the absence of any commercial or financial relationships that could be construed as a potential conflict of interest.
